# Discrimination in Middle Eastern and North African Americans predicts Worse Mental Health as Mediated by Sleep

**DOI:** 10.1007/s40615-024-02149-z

**Published:** 2024-09-03

**Authors:** Pedram Rastegar, Philip Zendels, Amy Peterman

**Affiliations:** Health Psychology PhD Program, University of North Carolina at Charlotte, 9201 University City Boulevard Colvard, Charlotte, NC 28223 USA

**Keywords:** Middle Eastern and North African American (MENA), Discrimination, Sleep, Mental health

## Abstract

Discrimination is a salient stressor linked with a variety of health outcomes including depression and anxiety among Middle Eastern and North African Americans (MENA). Among other minoritized racial and ethnic groups, sleep difficulties have been identified as potential mechanisms that explain the relationship between discrimination and mental health. However, this has not been explored within MENA samples. Thus, the focus of this study is to examine if two sleep measures (e.g., sleep disturbance and sleep-related impairment) mediate the relationship between discrimination and two mental health outcomes: depression and anxiety. We recruited 126 MENA adults from across the USA through Prolific. Findings revealed that sleep-related impairment fully mediated the relationship between discrimination and mental health outcomes, whereas sleep disturbances only partially mediated these outcomes. Results suggest sleep difficulties are one pathway that could explain the relationship between discrimination and mental health among MENA. Future work should continue to explore this relationship, as well as investigate discrimination and sleep as possible places of intervention to protect the health of MENA individuals.

Discrimination is a salient chronic stressor for many minoritized racial and ethnic groups and has numerous consequences for both physical and mental health [1–4]. A growing body of research has found that discrimination is associated with sleep difficulties [5] and that one pathway that links discrimination to adverse mental health outcomes is poor sleep [5, 6]. While less research has focused on Middle Eastern and North African Americans (MENA), research supports that discrimination is associated with a variety of poor health outcomes including depression, anxiety, and poor self-rated health among this group [7]. Furthermore, very little research has explored mechanisms that link discrimination to poor health among MENA. While research has found sleep difficulties as potential pathways from discrimination to poor health among diverse samples which included White, Black/African American, Latinx, and Asian [8, 9], this has not been tested using a MENA sample. Thus, the goal of this study is to examine the mediating role of sleep disturbance and sleep impairment between discrimination and two mental health outcomes: depression and anxiety.

## Biopsychosocial Model of Racism

Discrimination is associated with a variety of poor health outcomes, and one way to explain this is through the biopsychosocial model [2]. The biopsychosocial model broadly examines the interaction of biological, psychological, and social contexts on health [10]. This model states that chronic environmental stressors, such as discrimination, can lead to over-activation of the stress response [2]. Stress responses can disrupt physiological and psychological systems which can lead to poor health outcomes [2, 4]. While many minoritized racial and ethnic groups have been studied via the biopsychosocial model lens, one understudied group of individuals is those of MENA descent.

## Middle Eastern and North African Americans

MENA are broadly defined as individuals from countries such as “Algeria, Bahrain, Comoros Islands, Djibouti, Egypt, Iran, Iraq, Israel, Jordan, Kuwait, Lebanon, Libya, Mauritania, Morocco, Oman, Palestine, Qatar, Saudi Arabia, Sudan, Syria, Tunisia,” as well as “Turkey and Armenia” (pages 76 and 77) [11]. Some literature includes other countries, but typically MENA individuals are descended from people who lived in these countries in Northern parts of Africa to some southwestern parts of Asia [11]. While there is considerable heterogeneity across these countries, there are several important reasons for studying them as a group under the MENA umbrella. These include a range of shared cultural values, including the importance of religions [11–13], a collectivist mentality, strong family values, and the importance of honor [12–15].

It is important to examine the unique experiences of MENA using a within-group design as minoritized racial and ethnic groups do not have equal rates of health disparities. For example, studies have long shown that disparities in adverse sleep and mental health outcomes vary across minoritized racial and ethnic groups including among Black/African Americans, Hispanics, and Asian Americans [16–18]. These differences across health disparities could be tied to unique cultural backgrounds, socioeconomic circumstances, and mental health attitudes [16, 17, 19]. Exploring the unique differences in health disparities within minoritized racial and ethnic groups will help researchers understand who is at the most risk for adverse sleep and mental health outcomes. Additionally, this may aid in facilitating the most appropriate interventions based on cultural resources and strengths. Therefore, it is important to understand the unique risk factors of adverse sleep and mental health outcomes among MENA.

## Discrimination and Health

It has been well documented that discrimination is associated with higher levels of internalizing symptoms such as depression and anxiety among many different minoritized racial and ethnic groups including African Americans, Asian Americans, and Latinx Americans [20–22]. Further, discrimination is associated with a variety of poor physical health outcomes [1, 4].

Compared to other minoritized racial and ethnic groups, there is less research on the health effects of discrimination among MENA. However, there is still strong evidence that demonstrates discrimination is a significant chronic stressor [13] that is associated with various adverse health outcomes. For example, one study among MENA identified that discrimination was associated with poorer self-rated health, less happiness, and higher distress (including symptoms of depression and anxiety) [23]. Other studies provide evidence that discrimination is associated with psychological distress including depression and anxiety, as well as poor self-rated health [7, 24, 25]. Similarly, discrimination predicted poor self-esteem [26]. Overall, discrimination is a relevant and pervasive stressor for MENA and is associated with many negative mental and physical health outcomes.

Unfortunately, there is little research that focuses on the mechanisms that explain how discrimination is linked to poor mental health. The biopsychosocial model illustrates that discrimination can activate the stress response, and over time, this can disrupt physiological and psychological systems which can then lead to poor health outcomes [2, 4]. The biopsychosocial highlights various mechanisms that may explain how discrimination leads to the development of mental health outcomes.

## Importance of Sleep

One possible mechanism highlighted in the biopsychosocial model is sleep. Sleep is important for maintaining a variety of key aspects of health. Adequate sleep is required for optimal cardiorespiratory health, immune function, digestion and metabolism, growth and development, cognitive functioning, and mental health [27]. Within MENA populations specifically, sleep health can be used as a predictor for multiple physiological and psychological health outcomes [28]. Researchers have demonstrated that sleep facilitates the allocation of energy to necessary biological processes including immune functioning, cell maintenance, and neural plasticity [29]. Thus, poor sleep is related to a variety of adverse health outcomes such as mood disturbance; individuals experiencing sleep impairments are at greater risk for depression, anxiety, stress, and other mental health outcomes [30]. Fragmented or restricted sleep has been shown to interact with stress reactivity as well, leading to poorer physiological and psychological health [31, 32]. Slow-wave sleep has been linked to the inhibition of stress hormone secretion, activation of the parasympathetic nervous system, and overall reducing stress responses [33]. It is because of this that sleep may mediate the relationship between discrimination and mental health.

Many mental health outcomes including anxiety, depression, and stress are often comorbid with sleep disorders such as insomnia, where an individual may struggle to fall asleep or maintain consistent sleep throughout the night [34, 35]. In the framework of the biopsychosocial model, insomnia is likely both a predictor of mental health outcomes as well as a symptom of them. For example, many individuals with depression do not respond to antidepressants when they experience insomnia; however, they may respond better if they overcome symptoms of insomnia [36]. Additionally, treatment directed toward sleep issues, such as l (CBT-I), has been shown to improve depression, anxiety, pain, and chronic stress [35, 36].

## Discrimination and Sleep

Chronic stress, such as exposure to racism and discrimination, can disrupt sleep [34, 37]. Many studies have explored the relationship between marginalized groups and sleep outcomes, with disadvantaged populations often getting shorter durations and worse quality sleep [27, 38]. A variety of research has investigated other racial and ethnic groups and how their sleep may be impacted specifically [39, 40]. For example, racist experiences in healthcare settings were associated with greater sleep disturbance and daytime fatigue among racial and ethnic minorities [41]. Additionally, Hispanic American immigrants who experienced more perceived racism reported greater sleep disturbance issues [6]. Racist experiences may lead to stress, which can in turn lead to sleep issues through processes like loneliness [42]. However, experiences of discrimination as related to sleep remain a relatively unexplored topic among MENA samples. Additionally, exposure to racism has been associated with long-term sleep issues and depressive symptoms in African Americans [39]. Regardless of exposure to racism, racial and ethnic minorities are significantly more likely to experience unhealthy sleep, which is often associated with greater risk for mortality and comorbid conditions such as depression [39, 43].

Given observed relationships between discrimination and sleep as well as sleep and mental health outcomes, sleep may mediate the relationship between discrimination and mental health outcomes. Conceptually, sleep may explain the relationship between discrimination and mental health as sleep is important in regulating stress [33]. Chronic stressors such as discrimination can activate the stress response, leading to sleep disruption over time which then decreases the regulation of the stress response [33]. Heightened activation of the stress response then increases the risk of developing mental health outcomes [44]. In summary, sleep may play a role in explaining the relationship between discrimination and mental health outcomes through its role in the stress response system.

A few studies have explored this cascading relationship; data from adolescents from minoritized racial groups demonstrated a mediation effect of discrimination through sleep on mental health outcomes, including mood disorders, rumination, and somatic symptoms [5]. Additionally, studies have explored individual ethnicities’s experience with discrimination and how sleep mediates the relationship with depression in Hispanic Americans, finding a full mediation effect [6]. It is thus possible that sleep mediates the relationship between racist experiences MENA individuals face and their mental health outcomes.

The present study seeks to investigate the relationship between discrimination, sleep health, and mental health outcomes in a MENA sample. While there have been a few studies that have investigated how discrimination toward subsamples of MENA could lead to sleep and mental health problems [45], this study hopes to explore the mediating role of sleep between discrimination and mental health in a larger, more diverse sample. We decided to explore these relationships uniquely in both depression and anxiety given the different prevalence rates in the different mental health outcomes among MENA [46]. We predict that within MENA individuals, those who have perceived more discrimination experiences will report greater symptoms of anxiety and depression. Additionally, we predict that this relationship will be mediated by sleep disturbances and sleep impairments; those who have experienced more discrimination will report more sleep disturbances and sleep-related impairments, contributing to poorer mental health.

## Methods

### Participant Characteristics

This sample includes 126 MENA adults. The mean age of the sample was 26.72 (SD = 8.23; range 18–56), and the majority identified as women (61.9%). Most participants reported being second generation (69.8%) and identified as heterosexual (69.0%). In terms of country of origin, 14.21% reported Palestine, 13.11% reported Lebanon, 11.48% reported Iran, and 9.29% reported Egypt. Full sample details are reported in Table 1. As country of origin was left open to response, 14 participants (11.11%) also responded with non-MENA countries.

**Table 1:** Participant characteristics

	M	SD
Age (*n* = 126)	26.72	8.23
	*N*	%
Gender identity (*n* = 126)		
Woman	78	61.9
Man	37	29.4
Gender minority	11	8.7
Country of origin (*n* = 126)**		
Palestine	26	14.21
Lebanon	24	13.11
Iran	21	11.48
Egypt	17	9.29
Syria	15	8.20
Iraq	10	5.46
Morocco	9	4.92
Turkey	9	4.92
Armenia	8	4.37
Jordan	8	4.37
Libya	4	2.19
Israel	4	2.19
Saudi Arabia	3	1.64
Generational status (*n* = 126)		
1st generation	18	14.3
2nd generation	88	69.8
3rd and 4th generation	20	15.9
Sexual orientation (*n* = 126)		
Heterosexual	85	75.2
Sexual minority	28	22.2
Prefer not to disclose	3	2.4

^**^Adds up to higher *N* than sample as participants could choose more than one response

### Procedures

Participants were recruited through Prolific, a research software shown to have high data quality [47]. In order to be eligible, participants must have been over the age of 18, self-identify as being of MENA descent, have the ability to read English, and have been living in the USA at the time of data collection. To find participants who identify as MENA, we utilized Prolific’s ethnicity category, including the Middle Eastern, African, and Mixed categories. This ensured participants who may fit our existing definitions of MENA were included, even if they may not include themselves in the sample. Participants were asked if they identify as MENA, expressed their ancestry, and were compensated 15 cents for completion of this survey. Those who completed this, self-identified as MENA, and met the rest of the eligibility criteria were invited to take part in the full survey (approximately 25 min) and received an additional $4.25 for completion. Altogether, 203 participants identified as MENA, and 126 completed the full survey that comprises our study sample. Informed consent was obtained from all participants, and all procedures were approved by the Institutional Review Board of a southeastern US university.

### Measures

#### Demographics

Participants reported their age and self-reported their gender identity from a list of ten options including the opportunity to write in. Additionally, participants reported on their own or their family’s country of origin, and they identified their generational status by choosing one of the following options: first-generation, second-generation, third-generation, fourth-generation, or temporary resident. Lastly, participants reported on their sexual orientation by choosing one of ten options that presented sexuality as a spectrum. Participants who reported exclusively heterosexual or only incidentally homosexual were grouped together, while others were placed in a sexual minority category for the purpose of data analysis.

#### Subjective Social Status: National

Participants completed the McArthur Scale of subjective social status (SSS) [48]. Given that subjective socioeconomic status (SSS) is associated with better sleep quality, we included this as a control measure [38]. The SSS is a one-item measure that asks participants where they would place themselves on a ladder with the highest point of the ladder being individuals who are the best off in terms of education, money, and respect in their occupation within the country and the bottom of the ladder being individuals who are the worst off. Higher scores indicated higher perceived subjective social status. Participants were varied in their perceived subjective social status (Table 2).

**Table 2:** Descriptive statistics and correlations

	*M*	SD	1	2	3	4	5	6
1. Discrimination	1.98	0.83						
2. Subjective SES	5.30	1.72	− 0.21*					
3. Depression	13.03	6.89	.34*	− .19*				
4. Anxiety	9.70	6.24	.33**	− 0.12	.66**			
5. Sleep disturbances	23.62	8.51	.26**	− .23**	.56**	.43**		
6. Sleep impairment	24.18	8.35	.33**	− 0.13	.62**	.58**	.65**	
7. Sexual orientation	*N* = 95		.13	− .13	.26**	.33**	.16	.31**

*N* = 125. Sexual Orientation dichotomized where 0 = exclusively heterosexual or only incidentally homosexual

*p < .05, **p < .001

#### Sleep Disturbance and Sleep-Related Impairment

Participants completed the PROMIS Sleep Disturbance short form and Sleep-Related Impairment short form [49]. For sleep disturbance, it reported their sleep difficulties within the past week, including trouble falling asleep, staying asleep, and how restless their sleep was on a 5-point scale ranging from “not at all” (1) to “very much” (5). For sleep-related impairment, participants reported on the frequency that functions of everyday life were impaired on the same 5-point scale. Example items include having a hard time concentrating, having a hard time getting things done, and feeling irritable. Items were summed for each measure individually with higher scores meaning worse sleep disturbance and worse sleep-related impairment. These scales had excellent internal consistency in our study (disturbance α = 0.94; impairment α = 0.93). Additionally, confirmatory factor analyses deemed acceptable fits (sleep-related impairment *X*^2^ = 39.239, df = 19, *p* < 0.005, Chi/df ratio = 2.065, CFI = 0.974, RMSEA = 0.94, SRMR = 0.039) for sleep-related impairment as well as sleep disturbance (*X*^2^ = 39.599, df = 14, *p* < 0.001, Chi/df ratio = 2.820, CFI = 0.971, RMSEA = 0.123, SRMR = 0.041) [50]. CFAs were conducted in separate univariate models. Acceptable fit was reached after one error term was correlated with another for sleep-related impairment and after five error terms were correlated for sleep disturbance. Error terms are correlated due to the similar meanings among the items [51].

#### Discrimination

In order to assess for discrimination, we adapted the Brief Perceived Ethnic Discrimination Questionnaire Community Version (Brief PEDQ-CV) [52]. The original measure assesses exposure to discrimination due to race or ethnicity across the lifetime. However, since research suggests that religion and race/ethnicity are interwoven among MENA [12] as well as due to discrimination derived from Islamophobia, MENA may perceive the source of discrimination as due to their ethnicity/race or religion. To account for this potential, we reframed the questionnaire to ask about perceptions of discrimination due to ethnicity/race or religion. We then administered the 17 items of the Brief PEDQ-CV which assesses a variety of forms of discrimination such as exclusion, stigmatization, discriminatory experience at work/school, and discriminatory threat/aggression. Additionally, we adapted some of the questions to make them more relevant to MENA, such as including perceived discrimination from airport officials. Participants were asked how often these forms of discrimination happened throughout their lives on a 5-point scale ranging from “never happened” (1) to “happened very often” (5). Scores were averaged across all items to create an average lifetime discrimination score, with higher scores meaning more discriminatory experiences. This scale had excellent internal consistency in our study (α = 0.95). Additionally, confirmatory factor analyses were conducted and deemed acceptable fit *(X*^2^ = 270.247, df = 116, *p* < 0.001, Chi/df ratio = 2.330, CFI = 0.902, RMSEA = 0.105, SRMR = 0.0651). Acceptable fit was reached after three error terms were correlated.

#### Mental Health

Participants completed the 10-Item Center for Epidemiologic Studies Depression Scale (CESD-10) [53] and the General Anxiety Disorder Scale (GAD-7) [54]. Participants reported symptoms of depression and anxiety within the past week and 2 weeks, respectively, on a 4-point scale with higher scores representing more frequent negative mental health symptoms. Items were then summed with higher scores representing worse mental health on each scale. Since both scales have been well validated and are considered reliable measures of depression and anxiety among various minoritized racial and ethnic groups, confirmatory factor analyses were not conducted [55–57]. Lastly, both scales had excellent internal consistency in our study (CESD-10 α = 0.87; GAD-7 α = 0.92).

## Data Analysis

### Descriptive Analysis

All data were analyzed with SPSS Version 28. Descriptive analyses were run to ensure data were normally distributed and had acceptable variance. No study item had more than 3% missing, and therefore, corrections for missingness were not implemented. Pearson’s correlations were conducted to examine relationships between study variables.

We explored generational status, sexual orientation, and subjective social status as our covariates. We did not include gender as a covariate due to the lack of significant differences among focal variables between gender groups. In order to examine the necessity of including generational status, we conducted one-way ANOVAs to explore differences in our study variables by generational status. Since the third and fourth generations had very small response totals (*N* = 11 and 9, respectively) and prior ANOVAs between them showed similar results, we combined them into one group. Similarly, only one participant identified as a temporary resident, and since we believe their experiences would be most similar to first generation, we recorded this participant as first generation. Lastly, we conducted correlations to explore if sexual orientation and subjective social status correlated with our focal variables to determine if they would be appropriate covariates.

### Primary Analyses

In order to test for mediating effects, we utilized Process Macro Model 4 in SPSS. Bootstrapping was conducted to compute confidence intervals. To reduce model complexity and multicollinearity, we conducted separate models for each mediator (sleep disturbance and sleep impairment). Generational status, sexual orientation, and subjective social status were included in the models as covariates with 2nd generation and heterosexuals as the reference groups. In order to detect mediation, we examined a direct relationship between our predictors on sleep outcomes, a direct relationship between sleep outcomes on mental health outcomes, and an indirect relationship of both the initial predictors and the sleep outcomes predicting mental health outcomes to detect if the effects were diminished [58]. Mediation was detected if the confidence intervals of the indirect effect did not cross zero.

## Results

### Preliminary Results

Table 2 shows our outputs from initial descriptive and correlation statistics. Discrimination was significantly correlated with all focal variables, with those experiencing greater perceived discrimination also reporting being of a lower subjective SSS (*r* =  − 0.21, *p* = 0.020), having greater depression (*r* = 0.34, *p* < 0.001) and anxiety (*r* = 0.33, *p* < 0.001), and having more sleep issues (disturbances *r* = 0.26, *p* = 0.003; impairment *r* = 0.33, *p* < 0.001). Individuals who reported higher subjective SSS reported lower depression scores (*r* =  − 0.19, *p* = 0.038) and fewer sleep disturbances (*r* =  − 0.23, *p* = 0.010). Depression was strongly associated with anxiety (*r* = 0.66, *p* < 0.001) and both measures of sleep issues (disturbance *r* = 0.56, *p* < 0.001; impairment *r* = 0.62, *p* < 0.001). Anxiety was also associated with greater sleep disturbance (*r* = 0.43, *p* < 0.001) and sleep impairment (*r* = 0.58, *p* < 0.001), and both sleep issues were correlated in expected directions (*r* = 0.65, *p* < 0.001). Other effects are shown in Table 2. Additionally, we found a significant correlation between sexual minorities reporting greater depression (*r* = 0.26, *p* = 0.004), anxiety (*r* = 0.33, *p* < 0.001), and sleep impairment (*r* = 0.31, *p* = 0.001).

In order to determine if generational status should be included in the model, one-way ANOVAs were conducted to examine differences in study variables by generational status. First, homogeneity of variance was met for all study variables except anxiety. Therefore, one-way ANOVAs were conducted for all study variables except for anxiety. This revealed significant differences for generational status for depression, *F*(2, 123) = 5.37, *p* < 0.01, and sleep impairment, *F*(2, 122) = 5.04, *p* < 0.01. Tukey post hoc comparisons revealed participants identifying as first generation had lower levels of depression (*M* = 8.89, SD = 6.04) compared to second generation (*M* = 13.22, SD = 6.64) and third generation (*M* = 15.90, SD = 7.28). Similarly, Tukey post hoc comparisons revealed participants identifying as first generation had lower levels of sleep impairment (*M* = 18.78, SD = 8.19) compared to second generation (*M* = 24.78, SD = 7.80) and third generation (*M* = 26.45, SD = 9.21).

Since homogeneity of variance was not met for anxiety, a Welch *F-*ratio test was conducted. This revealed significant differences in anxiety by generational status *F*(2, 38.27) = 21.79, *p* < 0.001. Further, we used a Game-Howell post hoc test since homogeneity was not met. This revealed participants identifying as first generation had lower levels of anxiety (*M* = 3.67, SD = 3.79) compared to second (*M* = 10.54, SD = 6.11) and third (*M* = 11.40, SD = 5.66) generations.

When running the primary analyses, we ran the model both including the one temporary resident as a first-generation immigrant, as well as excluding them. Results did not differ by significant amounts between analyses, and as such, we elected to include the participants for better data representation.

### Results of Primary Analyses

#### Sleep-Related Impairment

Table 3 explores the results of our mediation analysis of whether perceived discrimination predicts mental health outcomes through sleep-related impairment. Our model predicting sleep-related impairment was significant and explained about 22% of the variance seen in the model. Perceived discrimination was a significant predictor, with those who experienced more discrimination reporting more sleep-related impairment (*b* = 2.77, *p* = 0.002). Looking at the effects of perceived discrimination and sleep-related impairment on depression, sleep-related impairment was a significant predictor. Perceived discrimination had both a significant indirect (*b* = 1.19) and total (*b* = 2.27) effect as indicated by their non-zero confidence intervals. Because a significant direct effect was not observed (CI of − 0.17 to 2.33), this indicates a full mediation effect; perceived discrimination affects depression through sleep-related impairment, according to this model. Information regarding the impact of the covariates on the model can be found in Table 3.^1^

**Table 3:** Mediation results of perceived discrimination of depression and anxiety through sleep-related impairments

	SRI *b* (SE)	SRI (*β*)	CESD *b* (SE)	CESD *β*	CESD TE *b* (SE)	CESD TE *β*	GAD *b* (SE)	GAD *β*	GAD TE *b* (SE)	GAD TE *β*
*R*^*2*^	.22**		.42**		.21**		.44**		.30**	
Constant	20.01 (3.22)**		2.02		10.61 (2.66)**		.89 (2.36)		7.23 (2.28)*	
PEDQ										
Direct	2.77 (.85)*	.27	1.08 (.63)	.13	-	-	1.11 (.57)	.15	-	-
Indirect	–	–	–	–	1.19 (.42)*	.14	–	–	.88 (.57)*	.11
Total	2.77 (.85)*	.27	–	–	2.27 (.70)*	.27	–	–	1.98 (.60)*	.26
SRI			.43 (.07)**	.52	–	–	.32 (.06)**	.43	–	–
Sexual minority	4.50 (1.74)	.22	.42 (1.27)	.03	2.33 (1.44)	.14	1.81 (1.14)	.12	3.22 (1.23)*	.21
SES	− .31 (.41)	− .07	− .32 (.30)	− .08	− .46 (.34)	− .12	− .14 (.26)	− .04	− .24 (.29)	− .07
1st Gen	− 4.83 (2.04)*	− .20	− 1.62 (1.48)	− .08	− 3.70 (1.68)*	− .19	− 4.57 (1.33)**	− .26	− 6.10 (1.44)**	− .35
3rd Gen	.14 (1.92)	.01	1.59 (1.37)	.09	1.65 (1.59)	.09	− .35 (1.22)	− .02	− .31 (1.36)	− .02

*N* = 122

*b* unstandardized effect size coefficient, *β* standardized effect size coefficient, *SRI* sleep-related impairment, *CESD* Center for Epidemiologic Studies Depression Scale, *TE* total effect, *GAD* General Anxiety Disorder Scale, *PEDQ* Perceived Ethnic Discrimination Questionnaire, *SES* subjective socioeconomic status, *1st Gen* first-generation immigration status, *3rd Gen* third-generation immigration status

*p < .05, **p < .001

We found similar results for anxiety as an outcome mediated through sleep-related impairment, also shown in Table 3; the initial model explained 44% of the variance with sleep-related impairment and first-generation status being significant predictors. The total effects model accounted for 30% of the variance in anxiety (*R*^2^ = 0.30,* p* < 0.001) with a significant indirect effect (*b* = 0.88) and total effect (*b* = 1.98) of discrimination given the non-zero confidence intervals. Again, these results demonstrate a full mediation effect, suggesting discrimination impacts anxiety again via association with sleep-related impairments.

#### Sleep Disturbance

Additionally, we explored similar mediation analyses of perceived discrimination on depression and anxiety through reported sleep disturbance, as shown in Table 4. In predicting sleep disturbance, our model was significant, accounting for about 15% of the variance. Information regarding the effects of covariates and their influence on the model are shown in Table 4. Perceived discrimination was a significant predictor (*b* = 2.05, *p* = 0.03), with individuals who experienced greater discrimination reporting more sleep disturbance. In predicting depression, 39% of the variance was significantly explained. Both perceived discrimination and sleep disturbances were significant predictors of depression in this model. When looking at total effects, our model accounted for 21% of the variance with perceived discrimination having significant indirect (*b* = 0.75) and total (*b* = 2.27) effects. These results suggest a partial mediation effect; perceived discrimination may lead to sleep disturbance, and both perceived discrimination and sleep disturbance contribute to depression as an outcome.

**Table 4:** Mediation results of perceived discrimination onto depression and anxiety through sleep disturbances

	Disturbances *b* (SE)	Disturbances (*β)*	CESD *b* (SE)	CESD *β*	CESD TE *b* (SE)	CESD TE *β*	GAD *b* (SE)	GAD *β*	GAD TE *b* (SE)	GAD TE *β*
*R*^2^	.15*		.39**		.21**		.37**		.30**	
Constant	24.37 (3.4)**		1.69 (2.82)		10.61 (2.66)**		1.89 (2.59)		7.23 (2.28)*	
PEDQ										
Direct	2.05 (.91)*	.20	1.52 (.64)*	.18	–	–	1.53 (.58)*	.20	–	–
Indirect			–	–	.75 (.37)*	.09	–	–	.45 (.25)*	.06
Total	2.05 (.91)*	.20	–	–	2.27 (.70)*	.27	–	–	1.98 (.60)*	.26
Disturbances			.37 (.06)**	.45	–	–	.22 (.06)**	.30	–	–
Sexual minority	1.38 (1.85)	.07	1.83 (1.28)	.11	2.33 (1.44)	.14	2.91 (1.17)*	.19	3.22 (1.23)*	.21
SES	− .94 (.44)*	− .19	− .12 (.31)	− .03	− .46 (.34)	− .12	− .03 (.28)	− .01	− .24 (.29)	− .07
1st Gen	− 3.70 (2.17)	− .15	− 2.35 (1.51)	− .12	− 3.70 (1.68)*	− .19	− 5.29 (1.38)**	− .30	− 6.10 (1.44)**	− .35
3rd Gen	1.76 (2.05)	.08	1.00 (1.41)	.05	1.65 (1.59)	.09	− .70 (1.30)	− .04	− .31 (1.36)	− .02

*N* = 122

*b* unstandardized effect size coefficient, *β* standardized effect size coefficient, *Disturbances* sleep disturbances, *CESD* Center for Epidemiologic Studies Depression Scale, *TE* total effect, *GAD* General Anxiety Disorder Scale, *PEDQ* Perceived Ethnic Discrimination Questionnaire, *SES* subjective socioeconomic status, *1st Gen* first-generation immigration status, *3rd Gen* third-generation immigration status

*p < .05, **p < .001

In predicting direct effects on anxiety, also shown in Table 4, our model significantly explained 37% of the variance. Perceived discrimination and sleep disturbance were both significant direct predictors. This suggests greater experiences of perceived discrimination and more sleep disturbance could lead to greater anxiety. In looking at the total effects, the model accounted for 30% of the variance observed. Perceived discrimination had a significant indirect effect (*b* = 0.45) and total effect (*b* = 1.98) in predicting anxiety; greater discrimination was associated with more anxiety. This again shows a partial mediation, as the effects of perceived discrimination contribute to anxiety both directly and indirectly through sleep disturbance. Additional effects of covariates are shown in Table 4.

## Discussion

In this sample of 126 MENA-identified participants, analyses confirmed our hypotheses about the mediating role played by sleep impairment and sleep disturbance in the relationship between discrimination and mental health. Based on previous research in other minoritized racial and ethnic groups, we predicted that MENA reporting perceived discrimination would experience more depression and anxiety and that this pathway would be mediated through sleep disturbance and sleep impairment. Across all models, there was a significant indirect effect of perceived discrimination on depression and anxiety. For our pathway exploring sleep-related impairment, the effects indicated a full mediation. Our pathway exploring sleep disturbance as a mediator found partial mediation, with a direct effect of perceived discrimination also contributing significantly to depression and anxiety outcomes. These findings suggest that perceived discrimination can lead to greater mental health issues, such as depression and anxiety. Additionally, these findings suggest sleep-related impairment is one likely cause for this pathway; perceived discrimination among MENA contributes to sleep-related impairment issues, which contribute to mental health issues, suggesting possible places for future research and intervention.

One notable finding was this pathway was only partially mediating in the sleep disturbance models, while there was full mediation in the sleep-related impairment models. This is contrary to some other findings, with studies exploring perceived racism in Hispanic Americans suggesting that sleep disturbance fully mediates the relationship between perceived racism and depressive symptoms [6]. The different findings observed could be a result of different measures used; the PROMIS scales feature a greater number of questions that are more specific to sleep disturbance and sleep-related impairment, while prior studies have used the Pittsburgh Sleep Quality Index, assessing sleep quality more broadly [6, 49]. Many aspects of sleep-related impairment may also be more noticeable throughout the day, including daytime sleepiness and problems functioning [49]. Past research on minority immigrants has found positive associations between excessive sleepiness and depression [59], and causes for sleepiness can sometimes be related to factors besides sleep disturbance [60], possibly explaining the differences observed. Lastly, it is important to acknowledge that many facets of sleep and mental health are bidirectional, with a variety of sleep problems being experienced by those with depression and a variety of depressive and anxiety symptoms being observed in those struggling to obtain adequate sleep [36, 61]. Given this, it is possible that the sleep disturbance pathway did not show full mediation due to the bidirectional effects of depression and anxiety impairing sleep as well.

Overall, our findings align well with the biopsychosocial model. Conceptually, discrimination activates the stress response which leads to sleep disruption such as sleep disturbance and sleep-related impairment. Sleep disruption demonstrated in our findings may lead to heightened activation of the stress response which then increases the risk of developing poor mental health outcomes as illustrated in our findings.

Interestingly, we found first-generation MENA had lower sleep impairment, depression, and anxiety compared to second-generation MENA in our regression models. Furthermore, ANOVAs revealed first generation had lower levels of sleep impairment, anxiety, and depression compared to both second- and third-generation MENA. This is consistent with the literature showing the first generation having better health including sleep compared to minoritized racial and ethnic groups who are US-born [62, 63]. Across the literature, multiple models have attempted to explain why first-generation immigrants tend to have better health. Some of these include the resilient immigrant model which states that the process of immigration favors individuals who are resilient and have better mental health [64]. However, there is criticism of this model, as this does not consider the context or factors in how an immigrant arrived in the USA as these could play a role in explaining health outcomes [65]. Alternatively, another explanation could be that aspects of one’s culture such as family support or religiosity may be protective [11]. This concept is sometimes referred to as the protective culture model [64]. Lastly, immigrants may appraise the country they immigrated to as more favorable compared to their country of origin which could lead to better well-being [65]. Future research should continue to explore how the immigration process may relate to mental health outcomes.

Lastly, we found that identifying as a sexual minority correlated with both depression and sleep impairment and predicted anxiety in our regression models. Identifying as a sexual minority is consistently linked with poor mental health across the literature [66] which could be due to further experiences of discrimination [67] as well as potential experiences of discrimination due to intersectionality of identifying as a sexual minority and MENA.

### Implications and Significance

Our findings are important within the context of a MENA sample. Little research has focused on the effects of discrimination on MENA. To our knowledge, no research has explored causal pathways or mechanisms that explain the relationship between discrimination and mental health among MENA. This research is the first to point to sleep difficulties (e.g., impairment and disturbance) as one potential pathway to explain the relationship between discrimination and mental health which replicates reports for other minoritized racial and ethnic groups [6]. This research could lead to meaningful interventions for clinicians. Ultimately, we see that many mental health outcomes are predicted by social factors such as racism and experiences of discrimination. Some of these mental health issues may be addressed in the short term by looking at improving initial biological and behavioral aspects of health, such as practicing better sleep hygiene and treatment for insomnia as a way to buffer against the negative effects of discrimination [28, 34]. Thus, if clinicians are working with MENA clients who have experienced discrimination, it is critical to assess for sleep impairment and sleep disturbance. Furthermore, it would be important to target clinical interventions to alleviate sleep difficulties. For example, introducing sleep hygiene strategies and CBTI-I to combat sleep disturbance could help alleviate mental health outcomes regardless of association with discrimination [35, 36]. Given past observed relationships between sleep and positive mental health outcomes, targeting sleep as an immediate, actionable measure could help reduce clinical issues broadly.

### Strengths and Limitations

This study has several strengths. Though MENA is a new ethnic category with a broad definition [68, 69], we were able to include a large number of people and found similar experiences among them in regard to our study variables. Investigating a MENA-only sample also highlights the unique discrimination experiences faced, with similar patterns emerging in other ethnic groups [6]. Beyond the inclusive definitions for MENA, we also used inclusive definitions for gender and other demographic characteristics. Participants were able to select from sexuality options beyond the binary possibly providing them more recognition. However, the wording we used may not have reflected all the identities of gender and sexuality of the participants. Providing more options and the opportunity for participants to self-report may promote more honest answers, allowing us to recognize potentially marginalized groups in research [70]. This study also operationalizes the multi-faceted nature of sleep and mental health [49]; by investigating sleep disturbance and sleep-related impairment as separate variables, we found similar but distinct effects. Lastly, we reached such a broad sample of individuals and had high-quality data through the use of Prolific. This provides our findings with more generalizability across MENA individuals throughout the USA and may represent higher quality data than similar online sampling methods [47].

However, this study is not without limitations. Though we sampled a broad definition of MENA across the country, we still had a relatively small sample size. Some of the countries of origin within our sample (e.g., Morocco, Turkey, Armenia, Jordan, Israel, Libya) were very underrepresented compared to others, which could contribute to their experiences not being representative of others. The small sample size also limited our ability to fully explore the data; for many of our analyses, we had to group individuals based on sexuality, generation status, and other categories that may have revealed more about the relationships between study variables.

The data also are cross-sectional; we cannot infer a directional relationship from the findings. It is possible that bidirectional relationships, such as mental health contributing to sleep outcomes, exist and help to explain the data [71]. Additionally, cross-sectional mediation analyses may lead to potentially biased estimates [72, 73]. Therefore, longitudinal research is needed. For example, a recent longitudinal study with Black Americans demonstrated that depressive symptoms mediated the relationship between discrimination and sleep but did not find a significant pathway from discrimination to sleep to depressive symptoms [39]. Future research should examine these longitudinal associations in MENA samples, as well.

Another limitation may be in our discrimination measure. To best capture MENA experiences of discrimination, we broadened our term to encompass perceived discrimination due to religious and ethnic identity. This is in part due to research suggesting that racial/ethnic identity and religious identity are interwoven among MENA [12]. However, one downside of this approach is that identities could be conflated. While our validity testing supports the use of this measure, future research may benefit from adopting an intersectionality approach such as by asking about perceived discrimination due to religious and racial/ethnic identity independently and together.

Finally, the limitations of self-report data should be acknowledged. There were some similarities between items measuring sleep-related impairment and items measuring mental health, again possibly confounding our results [49, 53, 54]. Given the nature of self-report data, it is also possible that participants were inaccurate in their responses or exaggerated symptoms experienced. However, all our measures had excellent internal consistency, suggesting this might not have been a large problem. Lastly, since our measures are all self-report, we may have higher correlations due to shared variance [74].

### Future Directions

A number of future directions emerged from our findings. First, it will be important to explore potential buffers for the effects of discrimination on sleep outcomes. Research suggests that among minoritized racial and ethnic groups, cultural resources are important coping strategies. Among MENA, religiosity and family are important sources of coping [11]. Measures of family connectedness and religiosity have been explored as buffers on the effects of discrimination on mental health outcomes [25]. It will be important to explore whether these cultural resources buffer the effects of discrimination on sleep outcomes. In general, exploring the within-group differences among MENA individuals should continue to be expanded upon in future research. Prior research has found that among other racial and ethnic identities (e.g., Black/African Americans, Asian Americans), there are unique differences in adverse sleep-related health outcomes that may be tied to unique cultural backgrounds, socioeconomic circumstances, mental health attitudes, and more [16, 17].

In addition to buffers, it will be important to explore the potential bidirectional relationship between sleep and mental health outcomes. Research suggests these relationships are reciprocal in nature [61]; however, this has not been explored in a MENA sample. It will be important to understand the directionality of how sleep difficulties relate to mental health outcomes. It is possible for example that discrimination may also lead to increases in anxiety and depression which then leads to increases in sleep difficulties [42]. Understanding this relationship will help clinicians create tailored treatment plans to assist MENA clients who are suffering negative consequences due to discrimination. Given the small sample size and novelty of the research question, other possible relationships could emerge. It is likely that the correlated nature of our sleep-related constructs could be further explored in future studies with a larger, more representative sample using a parallel or serial mediation pathway.

Lastly, it will be important for future research to explore other mechanisms that may explain the relationship between discrimination and sleep outcomes. For example, one potential pathway that could explain the relationship between discrimination and sleep outcomes is rumination. Individuals with depression often engage in rumination, which can contribute to worse insomnia [36]. Some research among minoritized racial and ethnic groups has also identified rumination as one potential pathway [75]. However, this has not been explored in a MENA sample. Exploring the potential mediating role of rumination could help elucidate the relationship between discrimination, sleep impairment, and sleep disturbance. As more work explores the relationship between sleep disparities among racial and ethnic minorities, future research should investigate societal change and policy intervention as well to minimize experiences of discrimination [34].

## Conclusion

MENA are an under-researched community with unique experiences that affect health behaviors and outcomes. This study explored how perceived discrimination experienced by MENA participants contributed to sleep issues and mental health outcomes. Notably, participants experiencing greater perceived discrimination also experienced worse depression and anxiety. This relationship was mediated by sleep; those who experienced greater discrimination got poorer sleep and suffered worse mental health outcomes. We observed a full mediation effect through sleep-related impairment and a partial mediation effect through sleep disturbance. These findings highlight the need to continue researching MENA individuals and their mental health outcomes, as well as the need for more targeted interventions. Focusing on improving sleep health could help improve other health outcomes in MENA populations who face discrimination.

## Data Availability

Data is available upon request.

## Footnotes

1 Due to concern that the sleep item on the depression measure might influence the findings, we also reran the analyses without the item, and the results were similar across both sleep-related impairment and sleep disturbance. Therefore, we kept the original analyses.

## Code Availability

Code is available upon request.
